# VMF3o: the Vienna Mapping Functions for optical frequencies

**DOI:** 10.1007/s00190-020-01385-5

**Published:** 2020-06-17

**Authors:** Janina Boisits, Daniel Landskron, Johannes Böhm

**Affiliations:** 1 Department of Geodesy and Geoinformation, TU Wien, Vienna, Austria; 2 Present Address: Federal Office of Metrology and Surveying (BEV), Vienna, Austria; 3 Present Address: Agricultural Public Authority Lower Austria, Hollabrunn, Austria

**Keywords:** Troposphere, Mapping functions, Horizontal gradients, SLR

## Abstract

The troposphere is considered as one of the major error sources in space geodetic techniques. Thus, accurate troposphere delay models are essential to provide high-quality products, such as reference frames, satellite orbits, or Earth rotation parameters. In this paper, a new troposphere delay model for satellite laser ranging, the Vienna Mapping Functions 3 for optical frequencies (VMF3o), is introduced. The model parameters are derived from ray-traced delays generated by an in-house ray-tracing software. VMF3o comprises not only zenith delays and mapping functions, but also linear horizontal gradients, which are not part of the standard SLR analysis yet. The model parameters are dedicated to a signal wavelength of 532 nm. Since some SLR stations operate also with other wavelengths, VMF3o provides a correction formula to transform the model parameters to any requested wavelength between 350 and 1064 nm. A test demonstrates that the correction formula approximates slant delays calculated at different wavelengths very accurately. The remaining error for slant delays at a wavelength of 1064 nm adds up to only a few millimetres at $10^{\circ }$ elevation angle. A comparison study of the modelled delays that are derived from VMF3o and ray-traced delays was carried out to examine the quality of the model approach. The remaining differences of modelled and ray-traced delays are expressed as mean absolute error. At $5^{\circ }$ elevation angle, the mean absolute error is only a few millimetres. At $10^{\circ }$ elevation angle, it is at the 1 mm level. The results of the comparison also reveal that introducing linear horizontal gradients reduces the mean absolute error by more than 80% for low elevation angles.

## Introduction

The effect of the neutral atmosphere, in the following referred to as troposphere, on electromagnetic waves causes a delay of the signal. This delay is one of the major error sources in space geodetic techniques. For techniques operating with microwave signals, a typical value for the hydrostatic component of the delay is 2.3 m in zenith direction for a station at sea level and average meteorological conditions (Nilsson et al. 2013). The non-hydrostatic component depends on the amount of water vapour in the troposphere and the delay in zenith direction can reach up to several decimetres in equatorial regions (Nilsson et al. 2013).

A common way to correct for troposphere delays is to estimate them as a further parameter, additionally to parameters such as station coordinates, satellite orbit parameters or Earth rotation parameters (ERP). Another approach is to apply a priori zenith hydrostatic delays (ZHDs), which are easier to model due to their high predictability, and to estimate only the non-hydrostatic component of the zenith delay.

In satellite laser ranging (SLR), which is operating on optical wavelengths, the ZHD is slightly larger compared to the ZHD in microwave techniques. However, optical wavelengths are less sensitive to water vapour resulting in a non-hydrostatic component of only a few millimetres in zenith direction. Since SLR has fewer observations available compared to other space geodetic techniques, such as Global Navigation Satellite Systems (GNSS) or very long baseline interferometry (VLBI), troposphere delays are commonly not estimated, but need to be modelled. Due to the rather small non-hydrostatic component, which is less predictable than the ZHD, troposphere delay models can be assumed to be more accurate for SLR than for microwave-based techniques.

The current approach for modelling troposphere delays in SLR is to determine the total delay in zenith direction and to project this delay to the elevation angle of the observation, subsequently, using an isotropic total mapping function (MF). More details to the currently used model are given in Sect. 2. This approach does not allow the consideration of horizontal asymmetries and includes no vertical information of the troposphere.

The Refraction Study Group [RSG, see Noll and Tyahla (2012)], launched by the International Laser Ranging Service (ILRS) in the year 2000, examined shortcomings in the previously used model by Marini and Murray (1973). The inadequate dispersion model of the mapping function was identified as the main error source resulting in errors at the centimetre level at $15^{\circ }$ elevation angle, depending on the wavelength. The study group also suggested the inclusion of horizontal gradients based on a study by Gardner (1977), who estimated gradient corrections at the order of 2 cm at $10^{\circ }$ elevation angle.

A study carried out by Hulley and Pavlis (2007) investigates the effects of horizontal refractivity gradients derived from ray-traced delays on SLR observations. They find that the annual mean of north–south and east–west gradients in absolute magnitude is at the level of a few millimetres at $10^{\circ }$ elevation angle and the maximum can reach up to 50 mm at some stations for the same elevation angle. Furthermore, they examine the effect of introducing horizontal gradients on observation residuals, which leads to an improvement of variance differences of roughly 10%. When applying a total correction derived from ray-traced delays, also including horizontal gradients, the variance differences are improved by up to 40% or more.

A different approach is presented by Drozdzewski and Sośnica (2018), who investigate the possibility of directly estimating horizontal gradients from SLR observations. Although the estimated gradients strongly correlate with estimated station coordinates in case of a low number of available SLR observations, the authors report a reduced formal error of unit weight when estimating horizontal gradients. The results are also compared to horizontal gradients derived from GNSS observations as well as horizontal gradients derived from numerical weather models. In a long-term analysis, the best agreement was found between SLR-derived gradients and the hydrostatic component of horizontal gradients derived from numerical weather models.

Previous work to the model presented in this paper was done by Boisits et al. (2018). A preliminary version of VMF3o provides zenith delays and MF coefficients derived from ray-traced delays on a global grid. Tests were carried out using SLR observations to the LAGEOS-1/2 satellites (Pearlman et al. 2019a, b) for the year 2005. Applying the VMF3o MF coefficients results in a reduction of observation residuals especially at low elevation angles. Boisits et al. (2018) also investigated the effect of applying not only VMF3o MF coefficients, but also the VMF3o zenith wet delay derived from ray-traced delays to SLR observations. This approach results in an even more distinct impact on observation residuals.

In a recent study, Drozdzewski et al. (2019) examine the effect of the Potsdam Mapping Function (PMF) (Zus et al. 2015) on SLR-derived products. In this study, PMF time series including linear as well as nonlinear horizontal gradients are generated and applied to 11 years of SLR observation data. The authors report a systematic effect on SLR-derived products and an improved consistency between pole coordinates derived from SLR observations and other space geodetic techniques. Regarding station coordinates, differences of up to 2 mm in the north component are found. Thus, the authors recommend to extend the current troposphere delay model for SLR by considering horizontal gradients.

This paper presents the first rigorous description of the final VMF3o model. The model parameters are based on ray-traced delays, as were the parameters of the preliminary version (Boisits et al. 2018). Since the grid interpolation is suspected to be an error source, site-specific VMF3o parameters are now provided. Additionally, linear horizontal gradients are introduced as standard. The zenith total delay (ZTD), the total MF, and the linear horizontal gradients are split into a hydrostatic and a wet component. This allows for numerous scientific investigations, such as estimating zenith wet delays only or estimating a constant offset of the zenith delay components. In this way, possible biases in zenith delays could be revealed or parameters, such as the currently used value for the $CO_{2}$ content in the atmosphere, could be revised. Furthermore, the procedure of estimating VMF3o parameters is in full consistency with Vienna Mapping Functions 3 (VMF3) (Landskron and Böhm 2017) and the discrete horizontal gradients model (GRAD) (Landskron and Böhm 2018) for space geodetic techniques operating with microwave signals. This consistency could play an important role when investigating inter-technique biases.

Section 2 of this paper gives an overview of the theoretical background and the current status of troposphere delay modelling for optical wavelengths. Section 3 describes the determination of VMF3o parameters as well as the derivation of a wavelength correction formula based on ray-traced delays at multiple wavelengths. Section 4 comprises a model validation and a summary of VMF3o products and their availability. Conclusions are provided in Sect. 5.

## Troposphere delay modelling in SLR

Electromagnetic waves, such as laser signals, experience a propagation delay when travelling through the troposphere. The delay in zenith direction is defined as (e.g. Mendes and Pavlis 2004)

1 $$\begin{aligned} \Delta L^{z} = 10^{-6} \int _{S}N \mathrm{d}z \end{aligned}$$

with the path *S* through the troposphere in zenith direction, *dz* in length units, and the total group refractivity of moist air $N = (n-1)\cdot 10^{6}$, where *n* denotes the total refractive index of moist air. The zenith total delay can be split into a hydrostatic and wet component as follows:

2 $$\begin{aligned} \Delta L^{z} = \Delta L^{z}_{h} + \Delta L^{z}_{w} = 10^{-6} \int _{S}N_{h} \mathrm{d}z + 10^{-6} \int _{S}N_{w} \mathrm{d}z \end{aligned}$$

where the subscripts *h* and *w* denote the hydrostatic and wet component of the delay $\Delta L$ and the refractivity *N*, respectively.

The troposphere delay at a given elevation angle, also referred to as slant total delay (STD), can be modelled as (e.g. Nilsson et al. 2013)

3 $$\begin{aligned} \Delta L(\varepsilon ) = \Delta L^{z}_{h} \cdot MF_{h}(\varepsilon ) + \Delta L^{z}_{w} \cdot MF_{w}(\varepsilon ) \end{aligned}$$

where also the mapping function is split into a hydrostatic component $MF_{h}$ and a wet component $MF_{w}$, both as a function of the elevation angle $\varepsilon$. Since the contribution of the wet component is very small compared to the hydrostatic part at optical wavelengths, the following expression is more common in SLR analysis:

4 $$\begin{aligned} \Delta L(\varepsilon ) = (\Delta L^{z}_{h} + \Delta L^{z}_{w}) \cdot MF(\varepsilon ) \end{aligned}$$

with $MF(\varepsilon )$ as total mapping function.

As recommended by the International Earth Rotation and Reference Systems Service (IERS) in the IERS Conventions 2010 (Petit and Luzum 2010), the hydrostatic and wet components of the troposphere delay in zenith direction are calculated following (Mendes and Pavlis 2004):

5 $$\begin{aligned} \Delta L^{z}_{h}= & {} 0.002416579 \frac{f_{h}(\lambda )}{f_{s}(\Phi , H)}P_{s}\end{aligned}$$

6 $$\begin{aligned} \Delta L^{z}_{w}= & {} 10^{-4}(5.316f_{nh}(\lambda )-3.759f_{h}(\lambda )) \frac{e_{s}}{f_{s}(\Phi , H)} \end{aligned}$$

where $\Delta L^{z}_{h}$ is the hydrostatic and $\Delta L^{z}_{w}$ is the wet zenith delay in metres, $f_{s}$ is a function of the station latitude $\Phi$ and the geodetic height of the station *H*, $P_{s}$, and $e_{s}$ denote the surface pressure and the surface water vapour pressure in hPa, respectively. The expression $f_{h}$ represents the hydrostatic and $f_{nh}$ the wet (non-hydrostatic) component of the dispersion equation as a function of the wavelength $\lambda$ in $\mu$m (see Mendes and Pavlis 2004).

Following Marini (1972), the recommended mapping function is expressed as continued fraction in a truncated form, as proposed by Herring (1992):

7 $$\begin{aligned} \hbox {MF}(\varepsilon ) = \frac{1 + \frac{a}{1 + \frac{b}{1+c}}}{\sin \varepsilon + \frac{a}{\sin \varepsilon + \frac{b}{\sin \varepsilon + c}}}. \end{aligned}$$

The coefficients *a*, *b*, and *c* are calculated following Mendes et al. (2002) and are given as a function of the station latitude $\Phi$, the geodetic height of the station *H*, and the temperature at the station $t_{s}$. In the following, this model for calculating the zenith delay and the corresponding MF will be referred to as Mendes–Pavlis, or short MP model.

As described above, the MP model is based on meteorological data records at the SLR station. This allows a very accurate estimation especially of the zenith hydrostatic delay (ZHD), where the vertical profile can be easily modelled using the surface pressure at the site.

However, the MP model assumes horizontal symmetry, which is a simplification of true conditions. First, the troposphere is thicker in warmer (equatorial) regions than in cold (polar) regions (Mohanakumar 2008). Second, the refractivity of the troposphere is lower at the thermal equator than at the poles (Gardner 1977). These effects result in systematic variations in the troposphere delay dependent on the azimuth angle. Other azimuthal asymmetries originate, e.g. from weather conditions such as storms, that cause local deviations from the hydrostatic equilibrium (Hauser 1989).

Atmospheric azimuthal asymmetries are commonly modelled following Chen and Herring (1997) by introducing linear horizontal gradients:

8 $$\begin{aligned} \Delta L(\varepsilon ,a) = \Delta L_{0}(\varepsilon ) + \hbox {MF}_{g}(\varepsilon )(G_{n}\cos (a) + G_{e}\sin (a)) \end{aligned}$$

where $\Delta L_{0}(\varepsilon )$ denotes the isotropic component computed using Eq. 3 and the second term on the right accounts for the anisotropic part, with the north–south component of the gradients $G_{n}$, the east–west component $G_{e}$, azimuth angle *a* and a dedicated mapping function $MF_{g}(\varepsilon )$. This mapping function can be expressed as:

9 $$\begin{aligned} MF_{g}(\varepsilon ) = \frac{1}{\sin (\varepsilon )\tan (\varepsilon )+C} \end{aligned}$$

where Chen and Herring (1997) find $C=0.0031$ for the hydrostatic and $C=0.0007$ for the wet component of the microwave gradients. This gradient model is commonly used in GNSS and VLBI, but did not become prevalent in SLR analysis.

## Development of a new troposphere delay model

This section presents the parameters of VMF3o and their derivation from ray-traced delays. VMF3o comprises zenith delays, mapping functions, and linear horizontal gradients for the hydrostatic and the wet component of the troposphere delay, as well as a set of coefficients to transform all VMF3o parameters to an arbitrary wavelength within the optical frequency range (see Sect. 3.4).

### Ray-tracing at optical frequencies

The ray-tracing software RADIATE (Hofmeister and Böhm 2017) is part of the Vienna VLBI and Satellite Software (VieVS) developed at TU Wien (Böhm et al. 2018). The determination of the delays is based on a 2D piecewise linear ray-tracing approach (Hobiger et al. 2008) using numerical weather models (NWMs) provided by the European Centre for Medium-Range Weather Forecasts (ECMWF). The NWMs that are used for deriving VMF3o parameters have a temporal resolution of 6h, a horizontal resolution of $1^{\circ }\times 1^{\circ }$ and a vertical resolution of 25 pressure levels (for a more detailed description of the ECMWF NWMs, see https://www.ecmwf.int/en/forecasts/datasets/set-i).

RADIATE was designed to determine ray-traced delays in the microwave range. In order to derive VMF3o parameters for SLR, a new option for ray-tracing at optical wavelengths was implemented. Since the majority of SLR stations operates at a wavelength of 532 nm (Noll and Tyahla 2019), this wavelength is used to generate optical ray-traced delays and deriving VMF3o parameters.

To determine ray-traced delays at any arbitrary location as well as any azimuth and elevation angle, a dense 3D refractivity field is generated based on the meteorological parameters of the NWM. Computing a hydrostatic and a wet refractivity field separately allows the determination of the hydrostatic and the wet component of the troposphere delay. In order to create optical refractivity fields, the equations for hydrostatic refractivity $N_{h}$ and wet refractivity $N_{nh}$ following Mendes and Pavlis (2004) are evaluated:

10 $$\begin{aligned} N_{h}= & {} N_{gaxs}\frac{T_{d}}{P_{d}}Z_{d}R_{d}\rho \end{aligned}$$

11 $$\begin{aligned} N_{nh}= & {} N_{gws}\frac{\rho _{w}}{\rho _{ws}} - N_{gaxs}\frac{T_{d}}{P_{d}}\frac{Z_{d}}{Z}\frac{e}{T}\frac{M_{w}}{M_{d}} \end{aligned}$$

where $N_{gaxs}$ is the group refractive index of dry air (Ciddor 1996), $T_{d}$ is the temperature of dry air, $P_{d}$ is the pressure of dry air, $Z_{d}$ is the compressibility factor of dry air, $R_{d}$ is the mean specific gas constant of dry air, $\rho$ is the density of moist air, $N_{gws}$ is the group refractive index of water vapour (Ciddor 1996), $\rho _{w}$ is the density of water vapour, $\rho _{ws}$ is the density of water vapour at standard conditions, *Z* is the compressibility factor of moist air, *e* is the water vapour pressure of moist air, *T* is the temperature of moist air, $M_{w}$ is the molar mass of water vapour, and $M_{d}$ is the molar mass of dry air. For the computation of $N_{gaxs}$ and $N_{gws}$ following Mendes and Pavlis (2004), the $CO_{2}$ content is set to 375ppm as described in the IERS Conventions (Petit and Luzum 2010) and the wavelength is set to 532 nm. Thus, the generated ray-traced delays are valid for optical signals at 532 nm wavelength.

### New mapping functions for SLR

Equation 3 forms the basis of the mapping functions of VMF3o. The hydrostatic and the wet mapping function are determined by their coefficients *a*, *b*, and *c* as described in Eq. 7.

Following the approach of VMF3, the *b* and *c* coefficients are estimated only once and not per epoch to ensure that the main parameters of VMF3o are well determined. The properties of the ray-traced delays generated for this purpose are listed in Table 1. Since low elevation angles are most sensitive in terms of separating atmospheric effects from other error sources, elevation angles of $15^{\circ }$ and below are used for the derivation of VMF3o parameters.

**Table 1:** Properties of the ray-traced delays generated to estimate *b* and *c* coefficients of the mapping functions of VMF3o

Property	Specification
Ray-tracing software	VieVS ray-tracer RADIATE
NWM data	ECMWF ERA-Interim
Horizontal resolution of NWM	$1^{\circ }\times 1^{\circ }$
Temporal resolution of NWM	Monthly mean
Time span	2001–2010
Horizontal resolution of ray-traced delays	$5^{\circ }\times 5^{\circ }$
Temporal resolution of ray-traced delays	Monthly mean
Wavelength of ray-traced delays	532 nm
Elevation angles of ray-traced delays	$5^{\circ }$, $7^{\circ }$, $10^{\circ }$, and $15^{\circ }$
Azimuth angles of ray-traced delays	$0^{\circ }$, $45^{\circ }$, $90^{\circ }$, $135^{\circ }$, $180^{\circ }$, $225^{\circ }$, $270^{\circ }$, and $315^{\circ }$

The *b* and *c* coefficients of the hydrostatic as well as the wet mapping function are estimated in a least squares adjustment using the following fit formula (Lagler et al. 2013), where *p* represents any of the $b_{h}$, $b_{w}$, $c_{h}$, or $c_{w}$ coefficients:

12 $$\begin{aligned} {\begin{matrix} p(doy) &{}= A_{0} \\ &{}\quad + A_{1}\cos \left( \frac{doy}{365.25}2\pi \right) +B_{1}\sin \left( \frac{doy}{365.25}2\pi \right) \\ &{}\quad + A_{2}\cos \left( \frac{doy}{365.25}4\pi \right) +B_{2}\sin \left( \frac{doy}{365.25}4\pi \right) \end{matrix}} \end{aligned}$$

where $A_{0}$ is the mean value, $A_{1}$ and $B_{1}$ are the annual amplitudes, and $A_{2}$ and $B_{2}$ are the semi-annual amplitudes. For this purpose, the temporal resolution of monthly mean values and the horizontal resolution of $5^{\circ }x5^{\circ }$ (see Table 1) is absolutely sufficient and the computation time is restrained to a reasonable length. The coefficients of the fit formula above are approximated using spherical harmonics (SH) up to degree and order 12. Thus, $b_{h}$, $b_{w}$, $c_{h}$, and $c_{w}$ can be computed as a function of station location and time. This allows to easily add sites to the regular processing of station-wise VMF3o parameters without recalculating *b* and *c* coefficients.

While the *b* and *c* coefficients capture only low frequency variations, $a_{h}$ and $a_{w}$ reflect short-term changes in the troposphere. The ray-traced delays used for estimating the *a* coefficients are generated with the properties listed in Table 2. The *a* coefficients of the isotropic mapping functions $MF_{h}$ and $MF_{w}$ are then determined by solving Eq. 7 for a and averaging over all azimuth angles. Unlike the *b* and *c* coefficients, the *a* coefficients are site-specific parameters to avoid potential error sources such as grid interpolation and height extrapolation.

**Table 2:** Properties of the ray-traced delays regularly generated to estimate *a* coefficients of the mapping functions of VMF3o

Property	Specification
Ray-tracing software	VieVS ray-tracer RADIATE
NWM data	ECMWF ERA-Interim + operational + forecast model data
Horizontal resolution of NWM	$1^{\circ }\times 1^{\circ }$
Temporal resolution of NWM	6 h
Time span	Starting 1990
Horizontal resolution of ray-traced delays	Station-wise (see Sect. 4.2)
Temporal resolution of ray-traced delays	6 h
Wavelength of ray-traced delays	532 nm
Elevation angle of ray-traced delays	$5^{\circ }$
Azimuth angles of ray-traced delays	$0^{\circ }$, $45^{\circ }$, $90^{\circ }$, $135^{\circ }$, $180^{\circ }$, $225^{\circ }$, $270^{\circ }$, and $315^{\circ }$

### Horizontal gradients for SLR

Generating ray-traced delays at several azimuth angles (see Table 2) allows not only the derivation of isotropic mapping functions, but also the determination of linear horizontal gradients. With the mapping functions obtained in Sect. 3.2 the isotropic component of the troposphere delay $\Delta L_{0}(\varepsilon )$ according to Eq. 8 can be calculated. When subtracting $\Delta L_{0}(\varepsilon )$ from the slant delays at each azimuth angle, residuals $\Delta L_{res}(\varepsilon ,a)$ are determined containing only the anisotropic part of the delays and Eq. 8 can be expressed as (Landskron and Böhm 2018):

13 $$\begin{aligned} \Delta L_{res}(\varepsilon ,a) = MF_{g}(\varepsilon )(G_{n}\cos (a) + G_{e}\sin (a)). \end{aligned}$$

The residuals can be formed for the hydrostatic as well as the wet component of the total delay. Subsequently, the north and east component of the hydrostatic gradients $G_{n,h}$ and $G_{e,h}$, and the wet gradients $G_{n,w}$ and $G_{e,w}$ are estimated in a least squares adjustment based on Eq. 13.

Typical values for the north and east components of horizontal gradients are several tenths of a millimetre, thus, causing azimuthal variations at the centimetre level when mapped to low elevation angles. For microwave signals, the hydrostatic and wet components of the gradients are at the same order of magnitude. For optical wavelengths, however, the wet component is significantly smaller compared to the hydrostatic component, as was already reported by Hulley and Pavlis (2007). Here, the horizontal gradients are split into a hydrostatic and a wet component in consistency with the general concept of VMF3o allowing more flexibility for scientific investigations.

### Wavelength correction formula

As described in Sect. 3.1, most SLR stations operate with laser signals at 532 nm, and hence, the ray-traced delays are generated using this wavelength for calculating the refractivity fields. Consequently, VMF3o parameters are valid for this specific wavelength. Since some stations also use other frequencies ranging from blue to near-infrared (NIR), the VMF3o model includes a correction formula to transform the parameters from 532 nm to other wavelengths. To derive such a formula, ray-traced delays at ten different wavelengths between 350 and 1064 nm are generated. The respective wavelengths and other properties of the delays are listed in Tables 2 and 3.

**Table 3:** Properties of the ray-traced delays generated to derive the wavelength correction formula of VMF3o. Only those different to the properties described in Table 2 are listed here

Property	Specification
NWM data	ECMWF ERA-Interim
Time span	2017-01-01 - 2017-12-31
Wavelengths of ray-traced delays	350 nm, 450 nm, 532 nm, 550 nm, 650 nm, 750 nm, 850 nm, 980 nm, 1050 nm, and 1064 nm
Elevation angles of ray-traced delays	$5^{\circ }$, $7^{\circ }$, $10^{\circ }$, and $15^{\circ }$

Based on these data, ten sets of VMF3o parameters (one for each wavelength) were calculated. When comparing the results for each wavelength to their reference at 532 nm, the frequency effect becomes most obvious for the ZHD. The differences between the ZHD at 532 nm and 1064 nm are at the 10 cm level, which cannot be neglected. The differences for the zenith wet delay (ZWD) are at the sub-millimetre level, causing deviations of several millimetres when mapped to low elevation angles. The differences for the mapping function coefficients and the horizontal gradients result in errors of up to several centimetres at low elevation angles. Hence, VMF3o parameters need to be corrected for observations at different wavelengths.

For the computation of the wavelength correction formula, each value was divided by its reference value at 532 nm and then averaged over all epochs and all stations. The result is one correction factor $p_{\lambda }/p_{532}$ per wavelength $\lambda$ for each VMF3o parameter *p*. The following fit formula was found to approximate the discrete values of the correction factors:

14 $$\begin{aligned} cf(\lambda ) = \frac{A}{\lambda ^{B}}+C \end{aligned}$$

where $\lambda$ is the target wavelength in nm and the coefficients *A*, *B*, and *C* are estimated in a least squares adjustment, independently for each parameter of VMF3o. The values found for *A*, *B*, and *C* are listed in Table 4. Applying the correction factor *cf* to any parameter $p_{532}$ of VMF3o yields the parameter $p_{\lambda }$ at the target wavelength $\lambda$:

15 $$\begin{aligned} p_{\lambda } = p_{532}\cdot cf(\lambda ) \end{aligned}$$

**Table 4:** Values found for the coefficients *A*, *B*, and *C* of the wavelength correction formula for each parameter of VMF3o

	*A*	*B*	*C*
$a_{h}$	992.41	1.9896	0.9964
$a_{w}$	10547.86	2.0306	0.9699
ZHD	48810.00	2.1730	0.9423
ZWD	772810.64	2.5042	0.8853
$G_{n,h}$	44970.00	2.1600	0.9431
$G_{e,h}$	74250.00	2.2495	0.9468
$G_{n,w}$	1.00	0.0000	0.0000
$G_{e,w}$	1.00	0.0000	0.0000

The coefficients for the wet gradients are chosen in a way that $G_{n,w}$ and $G_{e,w}$ do not change, since the coefficients are not well determined and the effect due to different wavelengths is negligible

**Fig. 1:**
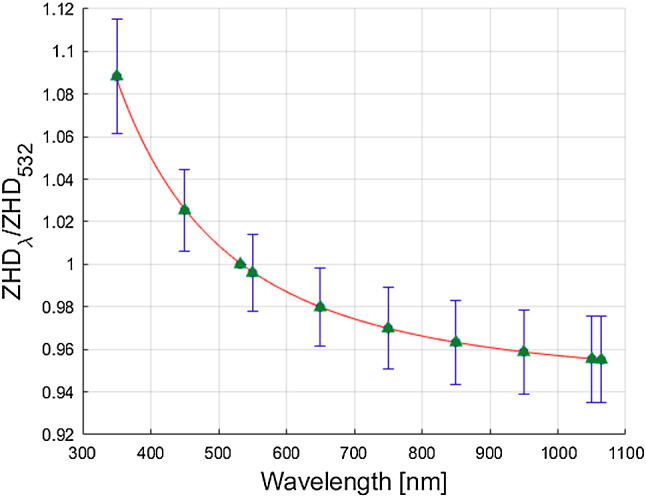
The blue dots (here covered by the green triangles) indicate the mean factor $ZHD_{\lambda }/ZHD_{532}$ for ten different wavelengths averaged over all epochs of the generated delays (see Table 3). The error bars are magnified by the factor $10^3$ for better visibility. The correction factor equals 1 for a wavelength of 532 nm. The red line illustrates the fitted curve using Eq. 14. The green triangles indicate the correction factors when using the dispersion equations of the MP model

**Fig. 2:**
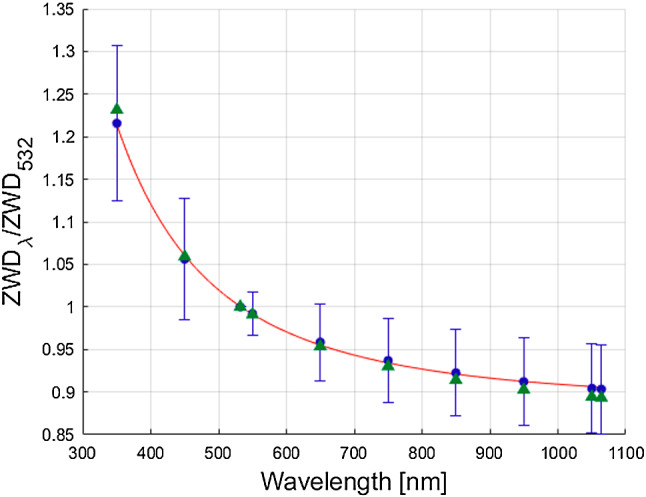
The blue dots indicate the mean factor $ZWD_{\lambda }/ZWD_{532}$ for ten different wavelengths averaged over all epochs of the generated delays (see Table 3). The factor equals 1 for a wavelength of 532 nm. The red line illustrates the fitted curve using Equation 14. The green triangles indicate the correction factors when using the dispersion equations of the MP model, differing only slightly from the values obtained from VMF3o time series

The discrete correction factors as well as the fitted curve using Eq. 14 are illustrated for the ZHD in Fig. 1 and for the ZWD in Fig. 2, exemplarily. Due to a limited number of decimal places in the output files, the correction factors of VMF3o parameters of small magnitude, such as the ZWD and the gradients, are less well determined compared to, e.g. the correction factors for the ZHD. Still, the wavelength dependency causes a clearly visible signal.

For verification, the obtained correction factors are compared to reference values based on the dispersion equations of the MP model (Mendes and Pavlis 2004). For this purpose, time series of ZHD and ZWD are calculated for the wavelengths listed in Table 3 using the MP model. Subsequently, discrete correction factors are computed in the same manner as described above. The values obtained from the MP model and the time series of VMF3o parameters, respectively, match very well, especially for the correction factors for the ZHD (see Figs. 1, 2).

To further examine the accuracy of the correction formula, the remaining errors between the corrected parameters using Eqs. 14 and 15 and the parameters obtained directly from ray-traced delays at the respective wavelength are formed. For a wavelength of 1064 nm, the residuals of the ZHD are below 0.5 mm and for the ZWD at the 0.1 mm level. The residuals of the coefficients $a_{h}$ and $a_{w}$ are mapped to a slant delay at $10^{\circ }$ elevation angle causing differences below 0.3 mm and 0.003 mm, respectively. The gradients are also mapped to a slant delay at $10^{\circ }$ elevation angle where they cause differences mostly smaller than 0.1 mm in direction of the largest amplitude. In total, the correction formula approximates the parameters directly retrieved from ray-tracing at the respective wavelength very accurately, with a remaining error of only a few millimetres at $10^{\circ }$ elevation angle, mostly due to the residuals of the ZHD.

## Validation and products of VMF3o

Section 4.1 examines the differences of the meteorological parameters pressure, temperature, and water vapour pressure, when (1) derived from NWMs and (2) measured at the site, as well as their impact on zenith delays and mapping functions. Section 4.2 presents a comparison of the delays computed using VMF3o, referred to as modelled delays, with ray-traced delays as a first validation of VMF3o. This allows to examine the performance of the model approach in general. Conclusively, all VMF3o products and their availability are listed in Sect. 4.3.

### Comparison of meteorological parameters

To get an idea of the accuracy of the pressure, temperature, and water vapour pressure (WVP) values from NWMs, they are compared to the meteorological measurements registered at SLR stations. The meteorological records are extracted from normal point files provided by the ILRS (Pearlman et al. 2019a). For this purpose, the monthly files containing the observations to LAGEOS-1 in the year 2017 are used. In the following, these values will be referred to as site values. The site-specific values of pressure, temperature, and WVP derived from NWMs are provided by VMF3o with a temporal resolution of 6h (see Sect. 4.3). For the comparison, they are calculated for the same epochs as the site values using a linear interpolation procedure. In the following, these values will be referred to as NWM values.

**Fig. 3:**
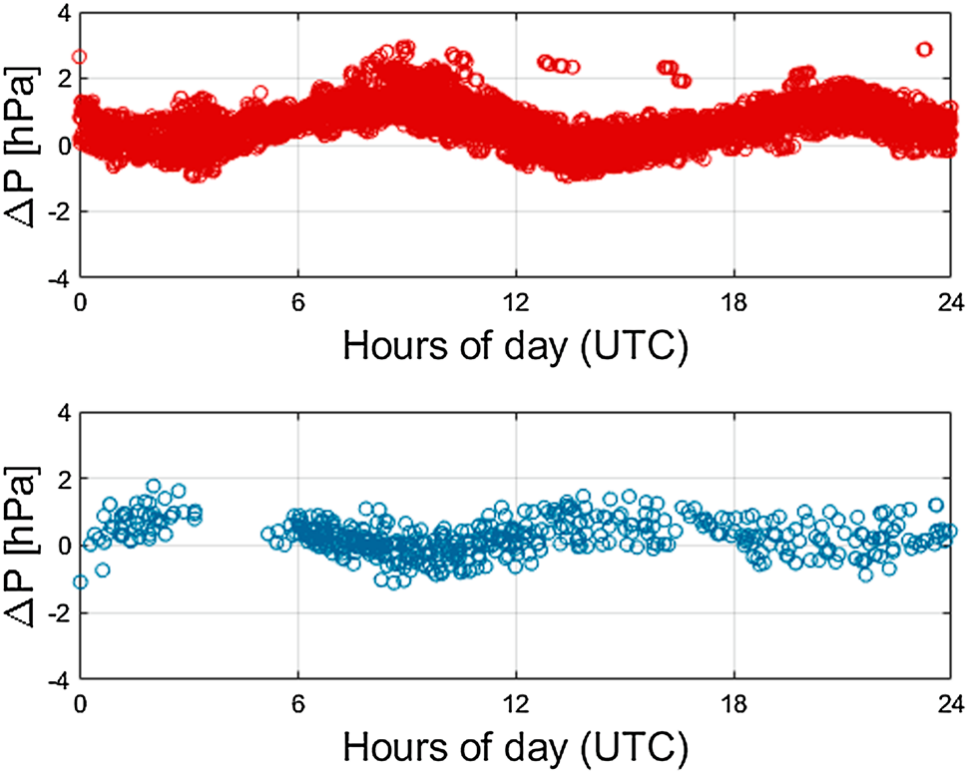
Stacked differences in pressure between site values and NWM values. Top: station 7090 (Yarragadee, Australia); bottom: station 7839 (Graz, Austria)

Figure 3 illustrates the pressure differences of site values and NWM values for the stations 7090 (Yarragadee, Australia) and 7839 (Graz, Austria). The residuals of each day in the year 2017 are stacked to get an idea of the daily variations. For both stations, a remaining semi-diurnal signal can be identified with an amplitude below 3 hPa. 1 hPa corresponds to an error of about 2.5 mm in ZHD. This signal does not decrease, when using a spline or cubic interpolation procedure instead of linear interpolation.

**Fig. 4:**
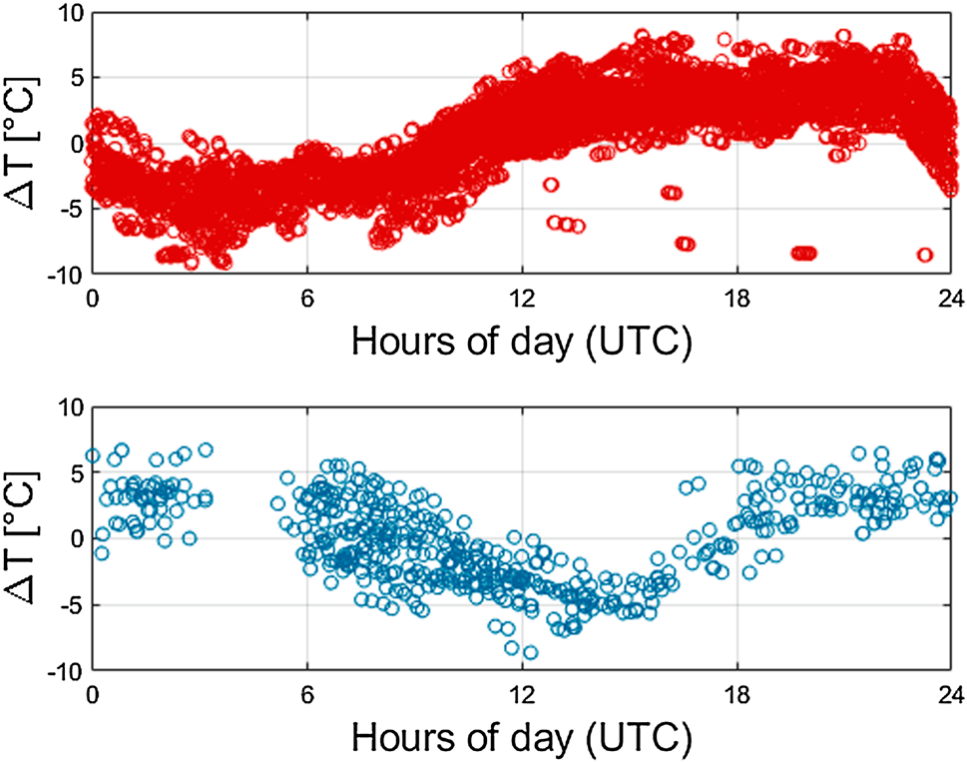
Stacked differences in temperature between site values and NWM values. Top: station 7090 (Yarragadee, Australia); bottom: station 7839 (Graz, Austria)

Figure 4 depicts the stacked differences in temperature for the stations 7090 (Yarragadee, Australia) and 7839 (Graz, Austria). Here, a diurnal signal with a maximum of about $9\,^\circ \hbox {C}$ can be identified. An error of $1\,^\circ \hbox {C}$ translates to deviations of approximately 0.6 mm in the STD at $10^{\circ }$ elevation angle. This signal also does not decrease, when using a spline or cubic interpolation procedure.

**Fig. 5:**
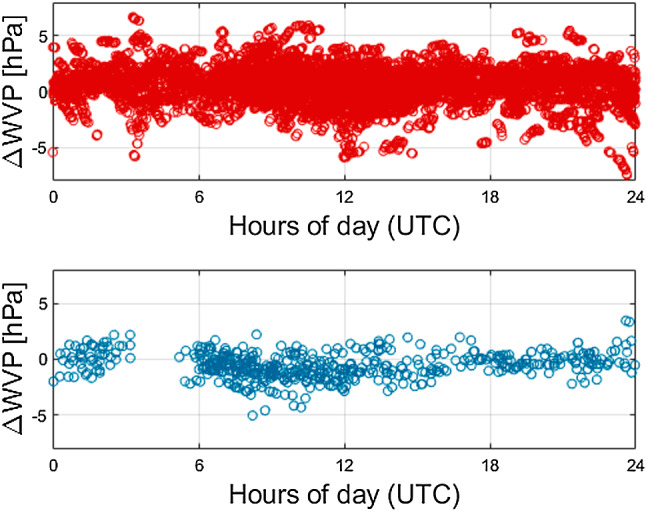
Stacked differences in WVP between site values and NWM values. Top: station 7090 (Yarragadee, Australia); bottom: station 7839 (Graz, Austria)

Figure 5 illustrates the stacked differences of WVP at the same stations. The results are also affected by the differences in temperature, when converting relative humidity to WVP. No clear periodic signals can be identified. The residuals range up to 5 hPa, which corresponds to approximately 1 mm in ZWD.

Table 5 provides the minimum and maximum values for bias and standard deviation of the differences when looking at all stations. Some values denoted as outliers are not listed in Table 5. This includes a pressure bias of 11.2 hPa at station 1879 (Altay, Russia), a pressure bias of 15.6 hPa at station 1889 (Zelenchukskaya, Russia), a pressure bias of 83.7 hPa at station 1890 (Badary, Russia), a temperature bias of $13.1\,^\circ \hbox {C}$ at station 1890 (Badary, Russia), as well as the consequential ZHD biases. A standard deviation of 14.5 hPa for the differences in pressure is found at station 7119 (Haleakala, Hawaii), which is also not included in Table 5.

**Table 5:** Minimum and maximum values of bias and standard deviation when looking at the differences of meteorological parameters at all stations

	Bias (min/max)	Standard deviation (max)
$\Delta$ P	$-$ 4.4/2.4 hPa	3.5 hPa
$\Delta$ T	$-2.6/5.8\,^\circ \hbox {C}$	$5.7\,^\circ \hbox {C}$
$\Delta$ WVP	$-$ 3.9/4.4 hPa	3.7 hPa
$\Delta$ ZHD	$-$ 10.7/5.7 mm	8.4 mm
$\Delta$ ZWD	$-$ 0.6/0.7 mm	0.6 mm

The differences in pressure P and WVP are also translated to deviations in ZHD and ZWD

Generally, meteorological data recorded at the station are expected to be more reliable. However, NWMs as additional source provide valuable information that can help to reveal biases originating in the meteorological sensors at the SLR sites. Furthermore, NWMs smoothen potential extreme local conditions, that are not representative for the whole column above the site, and do not depend on the daily rate of the sensors.

The results from the comparison indicate that the ZHD based on local records can be assumed to be more accurate. However, the computation of both, mapping functions and ZWD, based on ray-tracing could benefit from the vertical information provided by NWMs. A positive effect of ray-traced ZWDs was already reported by Boisits et al. (2018). Closer investigations on that issue still need to be carried out.

### Modelled delays versus ray-traced delays

A comparison of modelled delays and ray-traced delays was carried out to assess how precisely the VMF3o model approach represents ray-traced delays. VMF3o comprises zenith delays, mapping functions, and linear horizontal gradients for the hydrostatic and wet component of the delay. However, gradients of higher order are neglected. Landskron and Böhm (2018) find that second-order and third-order gradients further reduce the remaining differences, but only to a small degree. Furthermore, Drozdzewski et al. (2019) find that second-order horizontal gradients have only a small impact on SLR products and can be neglected, since SLR stations mainly operate during good weather conditions.

To investigate the remaining differences between the modelled and the ray-traced delays, 1460 epochs (4 epochs per day) of data in the year 2017 are used. The properties of the ray-traced delays generated for this purpose are listed in Tables 2 and 6. For the modelled delays, zenith delays, mapping functions, and linear horizontal gradients of VMF3o are applied and slant total delays at the azimuth and elevation angles according to Tables 2 and 6 are computed.

**Table 6:** Properties of the ray-traced delays generated for the comparison of modelled versus ray-traced delays. Only those different to the properties described in Table 2 are listed here

Property	Specification
NWM data	ECMWF ERA-Interim
Time span	2017-01-01 - 2017-12-31
Elevation angles of ray-traced delays	$5^{\circ }$, $7^{\circ }$, $10^{\circ }$, and $15^{\circ }$

The ray-traced delays serve as reference values in this study. For the comparison, mean absolute errors are formed. Table 7 lists the results for the slant total delay at station 7839 (Graz, Austria). At $5^{\circ }$ elevation angle, the mean absolute error ranges up to 5.5 mm and the effects of neglecting horizontal gradients of higher order are visible. However, at $10^{\circ }$ elevation angle, the remaining differences are only at the 1 mm level and the effect of higher-order gradients decreases rapidly with increasing elevation angle.

**Table 7:** Mean absolute errors of modelled minus ray-traced delays in mm averaged over all epochs at station 7839 (Graz, Austria)

	$0^{\circ }$	$45^{\circ }$	$90^{\circ }$	$135^{\circ }$	$180^{\circ }$	$225^{\circ }$	$270^{\circ }$	$315^{\circ }$
$5^{\circ }$	3.9	1.5	4.5	1.5	4.5	1.6	5.5	1.8
$7^{\circ }$	3.2	1.5	1.3	1.2	2.8	1.0	2.0	1.7
$10^{\circ }$	1.6	0.8	0.5	0.7	1.1	0.5	1.0	0.8
$15^{\circ }$	0.6	0.4	0.3	0.3	0.3	0.3	0.6	0.3

The values show the results for the slant total delay, where the hydrostatic component is responsible for the major part of the differences and the wet component contributes only on the sub-millimetre level. The results are listed for all four elevation and all eight azimuth angles separately

**Fig. 6:**
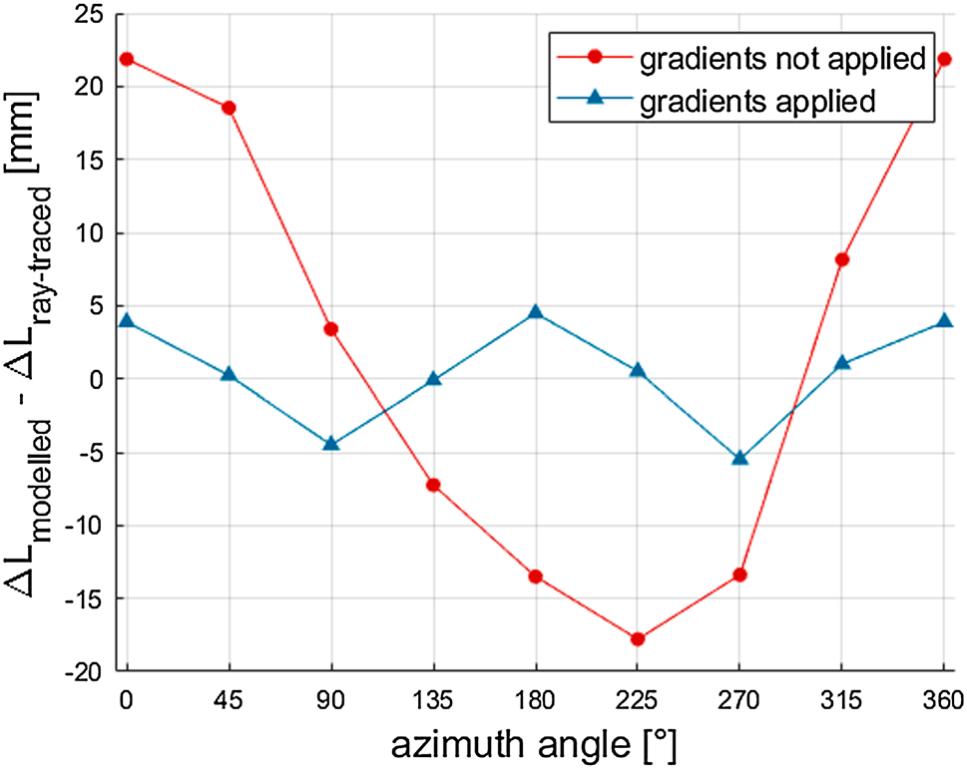
Mean error of total slant delays averaged over all epochs at station 7839 (Graz, Austria). The red dots depict the results at $5^{\circ }$ elevation angle when linear horizontal gradients are not applied. The blue triangles illustrate the results at $5^{\circ }$ elevation angle with gradients applied. The remaining error decreases from more than 20 mm to less than 5 mm when linear horizontal gradients are applied

Figure 6 illustrates the impact of applying gradients when modelling troposphere delays at low elevation angles. The figure depicts the results for station 7839 (Graz, Austria). At $5^{\circ }$ elevation angle, the mean differences between modelled and ray-traced delays range up to 20 mm and more when neglecting linear horizontal gradients. The mean error is reduced to less than 5 mm when applying gradients. These values roughly agree with the gradient corrections already found by Gardner (1977).

Looking at the mean values over all stations and all azimuth angles yields similar deviations. The results are listed in Table 8. At $5^{\circ }$ elevation angle, the mean absolute error is reduced from 18.2 to 3.1 mm, when applying gradients. This corresponds to an improvement by 83%. At $10^{\circ }$ elevation angle, the mean absolute error is reduced by 85% from 5.9 to 0.9 mm. When applying the mapping function of the conventional MP model for comparison, the differences to the ray-traced delays increase even compared to VMF3o with no gradients applied (see Table 8).

**Table 8:** Mean absolute errors of modelled minus ray-traced delays averaged over all epochs, all stations, and all azimuth angles in mm

$\varepsilon$	No LHG	LHG	MP	$\hbox {MP}^\text {RT}$
$5^{\circ }$	18.2	3.1	26.9	25.8
$7^{\circ }$	10.9	1.8	15.7	15.9
$10^{\circ }$	5.9	0.9	9.1	9.9
$15^{\circ }$	2.8	0.4	5.1	5.8

The values show the remaining error of slant total delays. The table lists the results for VMF3o with no linear horizontal gradients applied (No LHG) and for VMF3o including gradients (LHG). For comparison, the slant delays are also calculated using the MP model with meteorological data from the NWM (MP) as well as using the MP model only for the mapping function and the zenith delays obtained from ray-tracing ($\hbox {MP}^\text {RT}$)

### Availability of VMF3o products

VMF3o includes the following parameters:

- Zenith hydrostatic delay (ZHD) and zenith wet delay (ZWD),
- Coefficients $a_{h}$, $b_{h}$, and $c_{h}$ of the hydrostatic component of the mapping function according to Eq. 7,
- Coefficients $a_{w}$, $b_{w}$, and $c_{w}$ of the wet component of the mapping function according to Eq. 7,
- Hydrostatic north–south component $G_{n,h}$ and hydrostatic east–west component $G_{e,h}$ of the linear horizontal gradient model according to Eq. 8,
- Wet north–south component $G_{n,w}$ and wet east–west component $G_{e,w}$ of the linear horizontal gradient model according to Eq. 8,
- Coefficients *A*, *B*, and *C* of the wavelength correction formula according to Eqs. 14 and 15.

All products and auxiliary material can be found at the VMF Server under vmf.geo.tuwien.ac.at. The *b* and *c* coefficients can be calculated using the routine vmf3o_b_c.m and the SH coefficients stored in separate text files. The parameters $a_{h}$, $a_{w}$, ZHD, ZWD, $G_{n,h}$, $G_{e,h}$, $G_{n,w}$, and $G_{e,w}$ are provided in so-called VMF3o files (.vmf3o extension). Pressure, temperature, and water vapour pressure from the NWM are listed as additional information. VMF3o files are published once per day and contain four epochs corresponding to a temporal resolution of 6 h. A linear interpolation between these epochs is adequate (see Sect. 4.1). The parameters are calculated station-wise, so no grid interpolation is necessary. The station list includes all past and active SLR stations listed in the station coordinate file slr.ell. This file is regularly updated, if new stations are added to the latest version of the SLRF2014 SINEX file (Noll et al. 2018). Other stations, e.g. engineering sites, can easily be added manually and will be included in the processing from that day on.

There are three categories of VMF3o files:

- VMF3o_EI: VMF3o parameters are based on ray-traced delays using ECMWF ERA-Interim NWM data. These files are available beginning with the year 1990 until end of August 2019.
- VMF3o_FC: VMF3o parameters are based on ray-traced delays using the ECMWF forecast NWM. These files are available one day prior to the day of their validity.
- VMF3o_OP: VMF3o parameters are based on ray-traced delays using the ECMWF operational NWM. These files are available one day after the day of their validity.

All eight parameters provided in the VMF3o files can be transformed to a wavelength between 350 and 1064 nm. The coefficients *A*, *B*, and *C* to evaluate Eqs. 14 and 15 are provided in CF_ABC.txt.

## Conclusion

VMF3o is a new model for correcting troposphere delays in SLR. The model parameters are provided on the VMF Server on a daily basis. The model approach considers horizontal asymmetries of the atmosphere by introducing linear gradients. When comparing the modelled delays to ray-traced delays, the application of gradients reduces the mean absolute error by more than 80%.

Furthermore, the zenith hydrostatic and the zenith wet delays are derived from ray-tracing. Thus, they contain additional vertical information from the NWM compared to only using surface values of meteorological parameters. This is of special interest for the wet component, where the vertical profile is hard to predict using ground measurements.

With this approach, VMF3o aims for a further advancement of the current accuracy level of SLR products. The effect of troposphere delay models derived from ray-traced delays and including horizontal gradients on SLR products is already demonstrated by Drozdzewski et al. (2019). Furthermore, the processing scheme for VMF3o is analogue to the scheme for VMF3 (Landskron and Böhm 2017) and GRAD (Landskron and Böhm 2018), which are the follow-ups of the well-established VMF1 (Böhm et al. 2006) model for microwave techniques. Using troposphere delay models from a single source could help to overcome inter-technique biases. So far, however, no investigations on that issue have been carried out.

Currently, a more detailed validation of VMF3o is ongoing to assess the effect of VMF3o on SLR products. This work is done in close cooperation with the ILRS Associate Analysis Center at Wroclaw University of Environmental and Life Sciences. The results are expected to be published in the near future.

## Data Availability

VMF3o time series can be downloaded from http://vmf.geo.tuwien.ac.at/trop_products/SLR/. Additional scripts and data for the calculation of the *b* and *c* coefficients as well as the correction formula can be found at http://vmf.geo.tuwien.ac.at/codes/. For more details on the usage, see http://vmf.geo.tuwien.ac.at/readme.txt.
